# Flats and Flats

**Published:** 1893-04

**Authors:** 


### FLATS AND FLATS.

I'm dwelling in an up-town flat,
The rooms are few and small,
My nearest neighbor keeps a cat
That ranges through the hall,
Sometimes—but then what can he do
When doors are fast and windows too ?

In rear apartments, just athwart
The passage-way from mine,
A widow pumps in lusty sort,
An organ's wheezy chime,
And pours in song till hours are late,
The sorrows of her widowed state.

A parrot and a pug has she—
Uncompromising pair—
That never—never will agree,
Its feathers or its hair,
With barks and screeches, neither loath—
I wish Mephisto had them both !

The children, too, have divers plans
Of torturing the days;
The little urchins form in bands
Along the passage-ways
To sing and shout and madly rage—
These future Pattis of the stage!

And what with parrots, dogs and cats,
And children by the score,
I'm minded to abandon flats,
And seek them nevermore.
Else be it said beyond recall
That I'm the flattest flat of all.

LA CROIX.

